# Advancements in RNASeqGUI towards a Reproducible Analysis of RNA-Seq Experiments

**DOI:** 10.1155/2016/7972351

**Published:** 2016-02-10

**Authors:** Francesco Russo, Dario Righelli, Claudia Angelini

**Affiliations:** Istituto per le Applicazioni del Calcolo, CNR, 80131 Napoli, Italy

## Abstract

We present the advancements and novelties recently introduced in RNASeqGUI, a graphical user interface that helps biologists to handle and analyse large data collected in RNA-Seq experiments. This work focuses on the concept of* reproducible research* and shows how it has been incorporated in RNASeqGUI to provide reproducible (computational) results. The novel version of RNASeqGUI combines graphical interfaces with tools for reproducible research, such as literate statistical programming, human readable report, parallel executions, caching, and interactive and web-explorable tables of results. These features allow the user to analyse big datasets in a fast, efficient, and reproducible way. Moreover, this paper represents a proof of concept, showing a simple way to develop computational tools for Life Science in the spirit of reproducible research.

## 1. Introduction

RNA-Seq [1–4] is now the most widely used technology to study genome-wide gene expression and regulatory mechanisms in response to stress conditions or drug treatments and cell development as well as in the onset and progression of several diseases [5], including cancer. In particular, RNA-Seq experiments allow profiling an entire transcriptome under a condition of interest, detecting differences in transcriptional activities that can be associated with different physiological or pathological conditions and identifying and estimating isoform abundances as well as identifying novel genes or isoforms. The overall goal of RNA-Seq experiment is to understand which functional processes are significantly altered (either upregulated or downregulated) when comparing two or more conditions and, then, to identify the biological mechanisms regulating such changes.

Usually, RNA-Seq data analyses are complex and require the usage of several different tools to manipulate and process data, depending on the particular question the researcher is interested in (see [6, 7] for a review). When a reference genome is available, a typical analysis starts with the alignment of millions of raw sequences (i.e., short sequences of about 100 bp, often from paired-end libraries) collected for each sample by means of a mapping procedure (using TopHat [8], e.g.). For complex eukaryotic genomes such as human or mouse, the alignment files (usually in the so-called* bam* format) are quite big (about 2–5 GBytes per sample). In a typical experiment, researchers can produce from few units to tens of samples for a total amount that can reach tens or hundreds of GBytes. Moreover, with the decrease of experimental cost, such amount is expected to increase with fast rate.

Subsequently, the analysis proceeds with the gene expression quantification (i.e., it can be viewed both as a simple read counting over a list of annotated genes or as isoform quantification [2]). Then, the data has to undergo a series of preprocessing steps, which include filtering and normalization of the gene expression values aimed at making the samples comparable and removing different sources of biases.

To have a better insight into the biological process under study, a crucial step is the identification of differentially expressed (DE) genes across different biological conditions [7, 9, 10]. In this context, a researcher has to use one or more statistical tests that are able to assess whether observed differences in gene expression levels are more likely due to the differences in the biological conditions rather than to chance. A typical output of this step is a list of DE genes usually containing few hundreds of elements.

The final step of the analysis consists in the identification of pathways and functionalities significantly altered among conditions. Such point is crucial since it allows the biological interpretation of the analysis and it is usually known as Pathway and Gene Ontology analysis.

Several tools are available in the literature to carry out RNA-Seq analyses. Most of them operate as command-line (see, e.g., the Tuxedo pipeline in [11]). Unfortunately, the usage of command-line tools can be intimidating for those with a limited knowledge of programming languages. To this purpose, a series of web-servers and graphical user-friendly interfaces (GUIs) have been recently developed. For instance, the well-known web-platform Galaxy [12] has included several tools to build efficient pipelines for carrying out RNA-Seq data analysis and it represents one of the most efficient environments to handle big data over the cloud. The R package oneChannelGUI [13], originally developed for the analysis of microarray data, has been extended to handle RNA-Seq experiments and it is now a tool that combines several different functions for quantification and differential expression analysis. Analogously, RobiNA [14] and RNASeqGUI [15] are similar tools devoted to the identification of DE genes from RNA-Seq experiments. More recently, RAP [16] has been proposed as a cloud computing web-interface offering the possibility of creating modular analysis workflow. We refer to [17] for a comprehensive review of the available GUIs.

All those GUIs or web-platforms are easy to use and do not require a specific knowledge of a programming language. Therefore, they allow a nonexpert user to run complex and personalized analyses on the datasets of interest. On one hand, web-servers are more suited to build large pipelines that are automatically and completely executed on large amount of data; on the other hand GUIs are more suited for an interactive analysis of experimental data in which the researcher decides which step to perform on the basis of the inspection of preliminary results. However, the price to pay for this additional flexibility consists in the difficulty of keeping track of all actions performed while using GUIs [17]. Clearly, the latter point is considered a limit in terms of reproducibility of computational results.

In the last decade, we have seen a growing interest in the literature on the concept of* reproducible (computational) research* (RR in the rest of the paper) [18–21], motivated by the need to better improve the transparency of scientific publications and the knowledge transfer. In our opinion, RR is extremely important since it provides a way to inspect the correctness and authenticity of results presented in published papers. This feature consists in the possibility of reexecuting an entire analysis, or parts of it, of accessing all the details, of learning more about a particular study, or of replicating a study up to a certain point and then trying alternative analyses by using other methods (e.g., different normalization procedures and/or filtering procedures and/or DE methods and/or pathway types of analysis). The problem of the reproducibility of data analysis is of great relevance in the Life Science when the analyses are very complex, time consuming, and computationally demanding [22, 23].

In fact, to assure reproducibility, it is necessary to store all initialization parameters and codes of the methods used during an analysis. To date, the lack of reproducibility has constituted one of the main limitations of GUIs. However, in recent years, many different tools provide novel functionalities that support developers to build software (including GUIs) capable of keeping track of all actions performed while executing an analysis (see [24] for an overview).

In this work, we present the novel advancements we introduced in RNASeqGUI [15] with particular focus on the incorporation of the RR.

Since the first version of RNASeqGUI, we increased both the number of interfaces and the number of functionalities within each interface. We added the possibility of handling complex/multifactor designs (up to two covariates) by using several different DE methods and the possibility of conducting two different types of analyses for biological/technical replicates. We also introduced the possibility of performing the pathway analysis with three different methods, such as David [25], Graphite [26], and Gage [27], and of performing the Gene Ontology analysis with David and Gage interfaces. Each of these functionalities gives the possibility of querying some of the major pathway databases. In particular, via David and Graphite it is possible to query Kegg (http://www.genome.jp/kegg/), Reactome (http://www.reactome.org/), and Biocarta (http://cgap.nci.nih.gov/Pathways/BioCarta_Pathways), while Gage uses Kegg pathway database.

Moreover, in order to face the limit of the reproducibility of the analyses with GUIs, we incorporated the RR feature inside RNASeqGUI. As a result, all actions and steps are automatically recorded and visualized in a human readable report. This report integrates raw data, result tables, figures, and software code. Not only does the report contain detailed information about all the actions performed (along with all initialization settings), but also the code chunks are executed each time the user makes an action. Therefore, each code chunk corresponds to a part of the analysis and can be executed independently in R console. More precisely, the report is presented as* html *file in a human readable format, ready for submission as supplementary material of a publication or as piece of code in public repositories like* rpubs.com*. In a full RR spirit, each time the report file is generated, all the code chunks contained inside the report are executed again.

Clearly, the full reexecution might be very time consuming for both the authors and the readers of a publication, since the amount of data involved in RNA-Seq study can be very large. Moreover, a potential reader might not have the computational resources to run all the analyses. To address this limitation, we also implemented a feature in RNASeqGUI, called* caching *[28]. Even though this feature is widely used in many fields of Computer Science, from Internet browsers to smartphone apps, it is still not commonly used in Life Science. Caching constitutes a solution to speed up repetitive and computational expensive code chunks by using intermediate results stored in precomputed databases. In this way, a third-party user with small computational resources can either replicate some pieces of the analysis or execute an alternative analysis starting from a middle point in the report.

The paper is organized as follows. In Section 2.1, we describe the novel version of RNASeqGUI. In Sections 2.2 and 2.3 we explain how RR and caching have been implemented in our platform.  Section 2.4 explains how parallel computations are currently handled in RNASeqGUI.  Section 2.5 summarizes the main environmental requirements.  Section 2.6 describes how to extend RNASeqGUI by adding new functionalities. Finally, Section 3 concludes the paper and draws future development directions.

## 2. Material and Methods

### 2.1. RNASeqGUI

RNASeqGUI [15] is an open source graphical user interface, implemented in R, devoted to the analysis of RNA-Seq experiments. It requires the RGTK2 graphical library [29] to run and is freely available at http://bioinfo.na.iac.cnr.it/RNASeqGUI. Overall, RNASeqGUI integrates—in a unified platform—several of R packages commonly used in the analysis of RNA-Seq data.

RNASeqGUI works at two different levels at the same time. The first one is the* user level* composed of all the interfaces available to the user in order to analyse data, while the second one, the* reporting level*, is automatically executed while the user operates at the first level and regards the caching and reporting features. This new* reporting level *(not present in previous versions [15, 17]) automatically keeps track of all the operations performed at the* user level *by registering all the user actions and the input and output data and by creating the databases of the intermediate results. This second level makes the analysis reproducible and constitutes one of the main novelties of the new version of RNASeqGUI.

**Figure 1:**
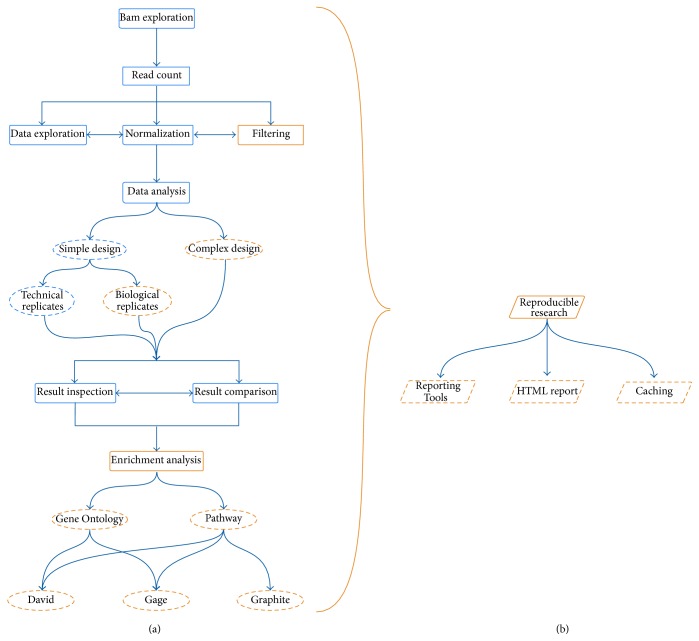
RNASeqGUI pipeline. Old features are represented in blue and the novel features are represented in orange. The boxes represent the software modules, while the ellipsis represents the modules functionalities. Panel (a) illustrates all the features the user can interact with, while panel (b) shows the reproducible research modules that work without user interaction. Note that panel (a) also illustrates a typical workflow to be executed during the analysis of RNA-Seq data experiments.

Figure 1 illustrates a typical RNA-Seq analysis workflow and represents a schematic view of the most important features available in RNASeqGUI. The old functionalities are represented in blue while the novel ones are represented in orange. Moreover, two levels, namely, panel (a) and panel (b), respectively,* user level *and* reporting level, *are illustrated.

#### 2.1.1. RNASeqGUI Main Interface

**Figure 2:**
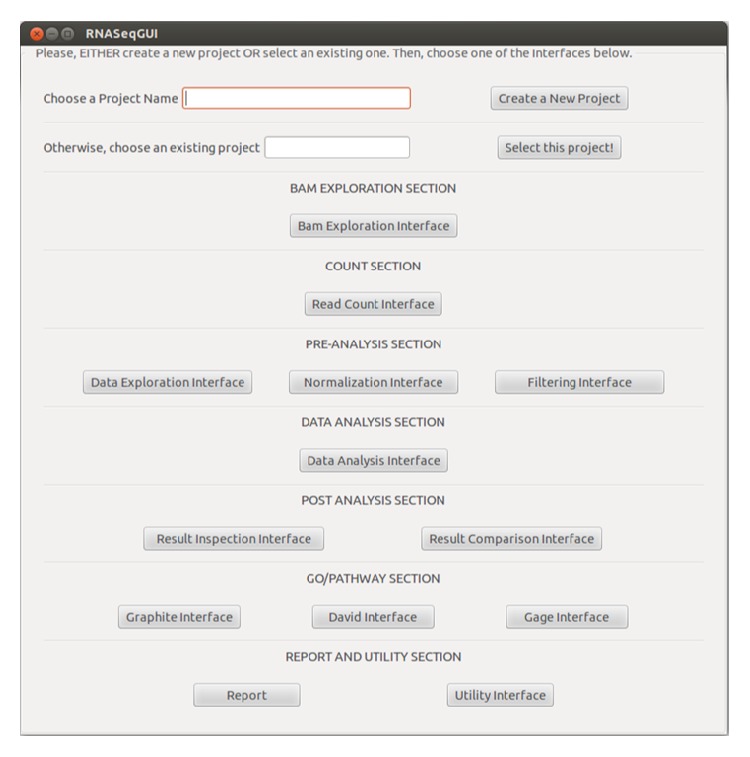
RNASeqGUI main interface.

The user interface of the novel version of RNASeqGUI (RNASeqGUI_1.1.0) is divided into seven main sections, as illustrated in Figure 2.

Each section is devoted to a particular step of the data analysis process and contains the access to one or more interfaces. RNASeqGUI is designed to represent a typical RNA-Seq analysis workflow that starts with the alignment file (in bam format). This approach is aimed at guiding the user through all the steps usually performed in an analysis. Clearly, the user is not obliged to access each section, but he can start from any section he wants and decide to skip some steps he considers unnecessary for the specific type of study carried on. As a consequence, RNASeqGUI results are very flexible for any type of usage.

Within each section or interface, the user can decide what is the most appropriate action to perform in the next step on the basis of the results obtained in the previous one. For instance, by looking at mean-difference plot (*MDplot*), density function (*Density or Qplot Density*), boxplot of counts (*Count Distr*), and scatterplot (*Plot All Counts*) generated in the* Data Exploration Interface* the user can decide if a normalization step is needed and also which type of normalization to perform.

In the current release, the first section covers files exploration of the alignment files (bam format). The second concerns the counting process of the mapped reads against a gene annotation file aimed at quantifying the gene expression levels. The third focuses on the exploration of count-data, on the normalization procedures, and on the filtering process, aimed at detecting and removing sources of biases. The fourth is about the identification of the DE genes that can be performed by several methods. Such a crucial section now includes also the possibility of handling complex/multifactor designs up to two covariates, as well as of using methods that can apply a suitable statistical hypothesis test in case of either technical or biological replicates (see Figure 1). Typical output of this section is the list of DE genes between conditions of interest.

The fifth section regards the inspection of the results produced by these methods and the quantitative comparison among them (via Venn diagrams). Using the interfaces available in this section it is possible to produce a wide series of graphical outputs such as Venn diagrams, volcano plots, fold change plots and histograms of *p* values, FDRs, and posterior probabilities. The novel sixth section regards the Gene Ontology and Pathway analysis (see Figure 1). The introduction of such a section allows a self-contained analysis and interpretation of the findings from a biological perspective.

Finally, the seventh section contains the button to generate the HTML report of the analysis executed (called* Report*) and* Utility Interface* that provides a series of useful functions for general purposes.

Therefore, the novel version of RNASeqGUI allows the user to conduct a complete analysis from the quality assessment of the alignment files to the Gene Ontology and Pathway analysis, deeply extending the range of applications with respect to previous versions [15, 17]. Moreover, thanks to some peculiar functionalities, like* Heatmap* in the* Gage* interface, it is possible to interpret the change in gene expression levels for a particular gene path of interest.

The user manual (available at http://bioinfo.na.iac.cnr.it/RNASeqGUI/Manual.html) constitutes a detailed description of all functionalities, with several suggestions and examples to guide the user through the analysis of RNA-Seq data.

Moreover, in the spirit of RR the novel version of RNASeqGUI keeps track of all actions made by the user and generates a final executable human readable report integrating data and tables of results and figure with executable code chunks. To the best of our knowledge, it is the first tool devoted to the analysis of RNA-Seq that combines the flexibility of an interactive* point&click* analysis with the tools that assure reproducibility [17].

#### 2.1.2. RNASeqGUI Usage

Each analysis must start with the creation or the selection of a project that refers to a specific experiment. In principle, the user should create a specific project for each dataset and for each workflow applied to such dataset. Then, by choosing the button corresponding to a desired step, it proceeds with the access to an interface necessary to configure all those parameters useful to perform the chosen step (for this task a button, called* “How to use this interface,”* helps the user to set them; however, more advanced information on the usage is provided in the user manual). After the configuration of all required parameters, the user must press the button corresponding to the action he wants to perform. Subsequently, in the R console several messages are displayed to inform the user about the progress of the execution (which can last for few seconds, several minutes, or hours, the latter for the functionalities in the* Read Count Interface*).

**Figure 3:**
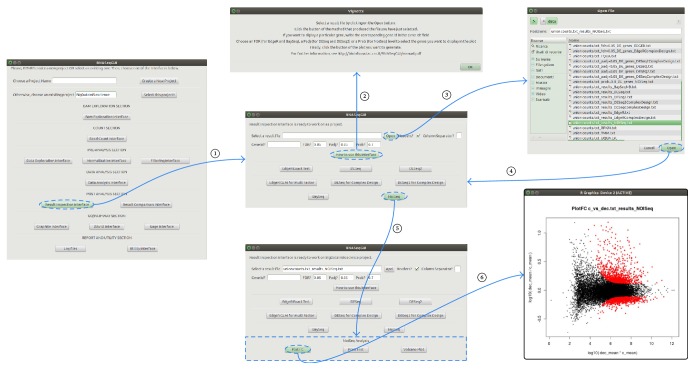
An example of execution flux for the* results inspection interface. *From the main interface, by clicking the* results inspection interface *button, a second interface opens. This interface is useful to inspect the results produced, by DE analysis. For each DE method, there is a dedicated button that opens a new box at the bottom of the interface. Such interface contains other buttons. We notice that each interface presents a “*How to use this interface*” button helping the user with the configuration of the parameters. After selecting results file (*NoiSeq results file *in this example), it is possible to use one of the buttons in the additional boxes, to make a graphical representation of the results (*PlotFC *in this example).

Figure 3 shows an example of interaction with the* Result Inspection Interface*, which helps to better understand how the software interfaces are structured.

After a job is executed, the results are presented to the user in a graphical form or, alternatively, the user receives the path where to access them. This second case shows up when the output consists of large tables.

As mentioned before, typical input data consists in a series of alignment files (in bam format) that can be obtained from the raw sequences using standard mapping procedures. Moreover, in order to quantify gene expression levels the user has also to provide a gene annotation file (in GTF format). The sections are aimed at guiding the user through the data analysis following flow-charts such as the one described in Figure 1. However, we set the sections to be as independent as possible. In this way, the user is not obliged to follow a predetermined flux of execution, but he is free to use each section without a preestablished order.

#### 2.1.3. RNASeqGUI Output

RNASeqGUI provides results of any action in graphical and/or table-formatted form. The first time the user creates a project, a specific folder, named as the project, is created in the* RNASeqGUI_Projects* root directory. In that folder the user will find all intermediate and final results of his analysis.

**Figure 4:**
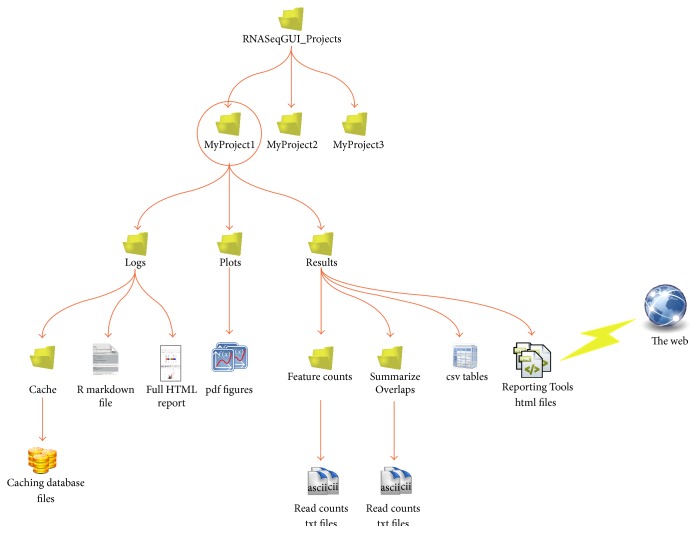
Output tree for the RNASeqGUI package.

The project directory contains three main directories named* Logs*,* results*, and* plots*, as illustrated in Figure 4.

The* Logs* folder contains all the files (*report.Rmd*,* report.html*,* report.md*,* report.txt,* and* sessionInfo.txt* files) reporting all the actions performed during the analysis (see Section 2.2) and a subdirectory named* cache* within the caching database files (see Section 2.3). Each database file is created when an action is performed to store the results obtained and the parameters used.

The* Results* folder contains all the tables produced during the DE analysis and the Pathway and Gene Ontology analysis. They are saved in* txt* and* tsv* (tab separated values) format. Moreover, when the Read Count Interface is used, a new subdirectory inside the* Results* folder is created to store the results of the specific read count function invoked (either* SummarizeOverlaps *from* Subread *package [30] or* FeatureCounts* from* GenomicRanges *package [31]).

**Figure 5:**
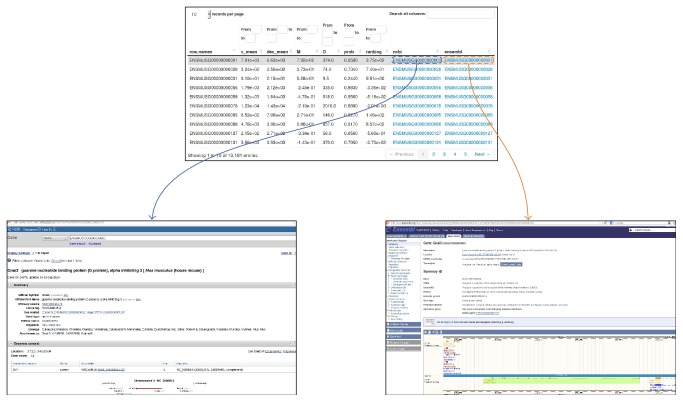
An example of HTML table using the ReportingTools package. By clicking on the gene of interest the author is redirected to well-known databases, such as NCBI or ENSEMBL.

In the* Results *folder, thanks to the* ReportingTools* package [32], most relevant result tables are also available in* html* format. Therefore, they can also be opened via a web browser (see Figure 5) and it is possible to interact with them. They can be filtered by values, sorted by using different column criteria. Moreover, it is possible to access available information of the genes by a single click. This action automatically redirects the user to two databases, such as http://www.ensembl.org/ and http://www.ncbi.nlm.nih.gov/, containing relevant biological information on the selected gene. Therefore, it is possible to retrieve information of biological interest in a fast and interactive way.

Finally, the* Plots* directory contains all the figures in* pdf* format, generated during the analysis.

### 2.2. Reproducible Research in RNASeqGUI

RR is the key aspect of the novel version of RNASeqGUI. By means of literate statistical programming novel internal module devoted to the reproducibility automatically keeps track of all lines of code corresponding to the actions performed by the user during the analysis, by writing (in the* Logs *folder) R markdown file, named* report.Rmd *(an example is given in Supplementary Material Figure  1, available online at http://dx.doi.org/10.1155/2016/7972351).

Each time an action is made by the user, RNASeqGUI registers it with a mark in the* Rmd* file, writing the executed R code. In this way, when the user clicks the* report* button (see Figure 2), the* report.Rmd *file is compiled and executes all the marks and the code lines and generates the HTML file named* report.html* (see Supplementary Material Figure  2).

Hence, this* report.html* contains all the information about the code lines used by RNASeqGUI plus all the initialization parameters and the input and output data. Such a report can be considered as a full detailed log file, written in human readable format, usable as supplementary material, containing executable code along with all initializations and printed results (plots, tables, arrays, etc.).

Therefore, not only does RNASeqGUI provide the open source code, but also all those lines do, which have been actually executed during a specific analysis. They are clearly reported as code chunks. These lines constitute complete and independent units of code that can be executed independently in R console without the need to install RNASeqGUI.

For instance, the Supplementary Material Figure  3 shows a scrap of the HTML report, which contains a code chunk used to produce a fold change plot (*PlotFC*). In this way, if a reader is interested in generating the same plot, he does not need to read the code of the entire analysis performed. It will be sufficient to copy and paste the code chunk for the particular step of interest inside R console, to generate the same plot. Finally, the user can compare the plot generated in this way with the plot depicted in the* report.html* to check whether they are identical. This can be done with all the code chunks inside the* report.html*.

### 2.3. Caching in RNASeqGUI

Another aspect of the RR is given by the possibility of fast reproducing and sharing of analyses and results via Internet.

In fact, when generating the report file, the execution of all code chunks used during the entire performed analysis can be very time consuming. Therefore, to face such issues we used* caching*: a strategy to store data into several objects in order to retrieve them in a faster and secure way.

**Figure 6:**
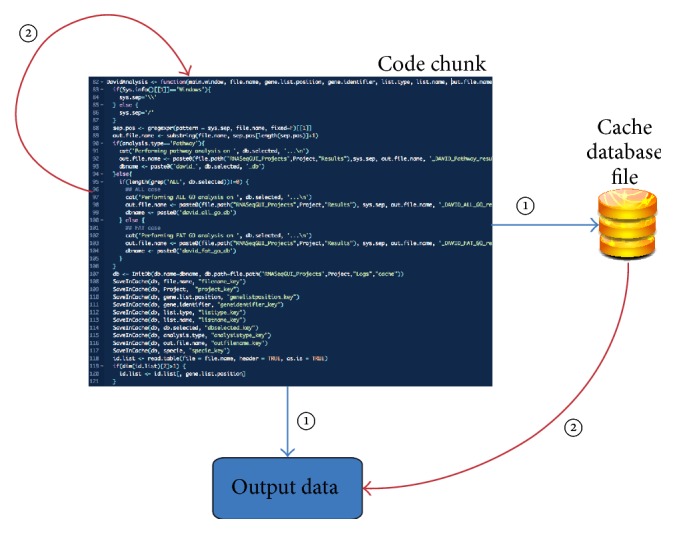
A typical execution flux involving the caching procedure. During step 1, a code chunk is executed producing output data and caching database file within input/output data. During step 2, when the same code chunk is executed, the output is drawn from the cache database.

Figure 6 represents a typical execution flux involving the caching procedure. During step 1, a code chunk is executed producing output data and caching database file within input/output variables. During step 2, when the same code chunk is executed, the output is drawn from the cache database file.

There are lots of R packages useful for* caching *[33–35]. We choose* filehash *[36], since it better fits our needs and storage idea. We wrapped some of its functionalities in order to implement, in the novel version of RNASeqGUI, a caching system to create a set of cache database files, stored in the* Logs/cache *folder, for each analysis flux (project) of RNASeqGUI. In this way, each function, when executed, generates a cache database file within the input/output variables and some partial computation data. These files are useful during the RNASeqGUI report generation.

**Figure 7:**
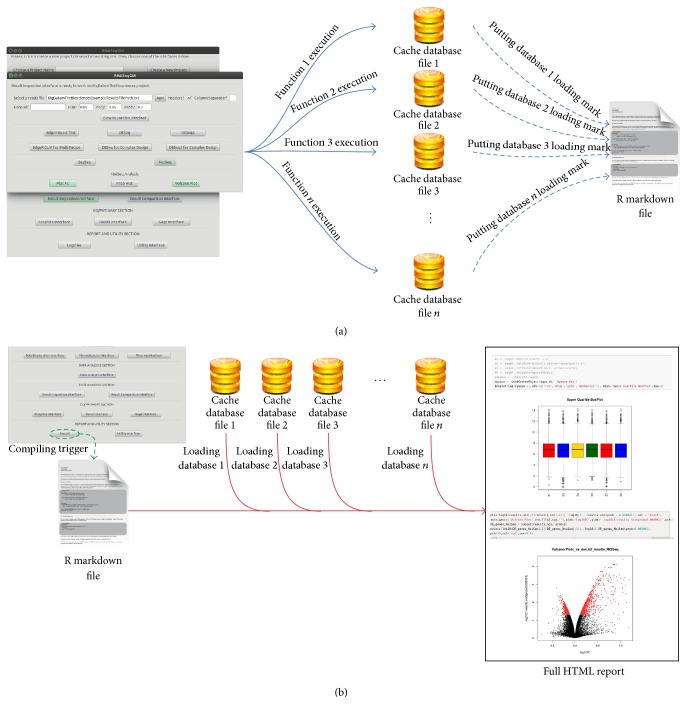
Schematic illustration of caching in RNASeqGUI. Panel (a) represents the caching file creation process. For each button of RNASeqGUI, one caching database file is created and a mark in the R markdown file is inserted, for its future load. Panel (b) represents the loading process during the report creation. Once the* html* button (in the* log files* section of RNASeqGUI) is selected, the R markdown file is compiled and data in the caching file are loaded to speed up the creation of the entire report.

Indeed, after the execution of each code chunk, RNASeqGUI generates a mark for it in the R markdown file (cf. Section 2.2) and a cache database file, traced in the R markdown file (see Figure 7(a)).

In this way, during the report generation (activated by the* report* button in the main interface) the data are loaded from the cache database file, speeding up the entire process (see Figure 7(b)), instead of reexecuting the entire code written in the* report.Rmd *file.

Moreover, in a complete spirit of transparency the user can share these files via Internet making it possible to reproduce the same analysis without complication of data research and manipulation.

In other words, caching makes all the intermediate results available in order to check them separately and to be used as starting points for different analyses. As a consequence, the implementation of caching allows the user to run in a more efficient way different types of analyses on the same dataset and to easily modify an analysis while still preserving reproducibility.

However, when sharing cached data through Internet, reproducibility might be limited unless both the raw data and the code needed to generate cached data are released.

To better understand how caching is implemented in RNASeqGUI, in Supplementary Material Figure  4 a scrap of the HTML report file is represented. To check the execution flux and to speed up the report compilation at the same time, both the commented code used to generate the cached data (in the blue parenthesis (A)) and the code used to load cached data (red parenthesis (B)) are reported. In Supplementary Material Figure  4(B) the result of the upper quartile normalization, stored in the* uqua.db *object, is loaded via the function* LoadCachedObject*. In this way, to check if the cached object is correct, a third-party user is able to generate the cached data by uncommenting the code reported in Supplementary Material Figure  4(A) that was used to produce the* uqua.db object*.

Furthermore, even if some code chunks are very fast to be generated (few seconds), it would be better to cache them as well, since during the generation of the HTML report, without them, all the code chunks are reexecuted and the overall process could last for several minutes.

To allow a better management of the entire data analysis and an automatic way to keep track of the computational protocol used for analysing a specific dataset, we combined a human readable report, within the code chunks, and caching in RNASeqGUI.

We stress that each execution of RNASeqGUI is linked with the name of the project chosen by the user and the name of the input file used. All the settings are saved in the report. Therefore, the user will keep track of all changes of the input parameters used. However, if a user changes the parameters within the same project and with the same input file then the cashed object will be overwritten along with the previous result file. To avoid such problem, one should create a single project for a specific workflow. Therefore, if a user wants to try two or more different settings of the same method then he has to create one project for each setting.

### 2.4. Parallel Computing in RNASeqGUI

Another crucial aspect, while working with large amount of data, is the computational cost required to complete each job. In particular, when working with large alignment files from RNA-Seq experiments, the most computational demanding step consists of the read counting process (i.e., the quantification level of each gene in each sample). To handle such process in a reasonable amount of time also on standard desktop, we used parallel computing within the R environment.

There are several packages that help to implement parallel computing in R, like* doparallel* [37] combined with* foreach* [38] and* snow *[39]. In RNASeqGUI we used* BiocParallel *[40], a package allowing parallel evaluation for* Bioconductor* [41] objects. We chose this package for its multiplatform portability and since it is optimized to work on bam files.

We tested the parallel computation by using two example datasets, one composed of six bam files of a cell culture from* mouse* (*mouse dataset*) and one consisting of seven samples of a cell culture from* Drosophila melanogaster *(*Drosophila dataset*), published in [42]. The* mouse dataset* has a total amount of data of about 38.4 GB and approximately 572 million reads, while the* Drosophila dataset* has a total of approximately 360 million reads for about 11.2 GB.

For the test we used two machines with R version 3.1.2: one desktop personal computer and one node of a cluster. The desktop PC is configured with an Intel I7-4790K@4.00 GHz running Ubuntu 14.04, while the cluster node is equipped with 12 cores of Intel Xeon X5650@2.67 GHz, running CentOS release 6.5.

As shown in Table 1, the computational time is drastically reduced when we made use of parallel computing, both on desktop PC and on cluster node.

On the rows of Table 1 the times in seconds for the tested datasets are reported, using the* SummarizeOverlaps* method of the* GenomicRanges *package [31]. The columns are the computational times, measured with and without parallel computing, on each machine, using 8 cores on the desktop PC and 12 cores on the cluster.

### 2.5. Installation and Environmental Requirements

RNASeqGUI is designed as a desktop application and requires a machine equipped with at least 8 GB of RAM. The novel version 1.1.0 successfully runs with R v3.2.2 and Bioconductor v3.2 with all major operative systems such as Linux, Mac OS X Yosemite, and Windows. Its functionalities work both on complex eukaryotic genomes (e.g.,* human* and* mouse*) and on simpler organisms (e.g.,* Drosophila melanogaster*). The installation procedure and the additional requirements (specific for each operative system) are detailed in the user manual, available at http://bioinfo.na.iac.cnr.it/RNASeqGUI/Manual.html. It is also possible to use RNASeqGUI (v 1.1.0) on a cluster environment. To start RNASeqGUI on the cluster, we simply used the command* ssh -X user@clusterhostdomain* and running RNASeqGUI in R shell as described in the manual. In this way, it was possible to use RNASeqGUI in remote mode from a computer running the* X unix window system*, making the data present on the cluster directly accessible by RNASeqGUI.

### 2.6. Extensibility

One of the most appealing features of RNASeqGUI regards the fact that it is relatively simple to add a new functionality. In fact, the steps necessary to add the new button (i.e., functions) are only three.

Firstly, the user has to write his own function, putting it in an appropriate R source file. After that, he has to write the code to create the button in the selected interface section, and, finally, he has to create the code to bind together the function and the button. The user manual explains through an example how the latter two steps can be performed.

As a consequence, in the spirit of open source, the user is allowed not only to include de novo developed functions, but also to use already developed packages in order to extend the features of RNASeqGUI. However, we note that the new method added by a user will not possess the reproducible research and caching features straightforwardly. Consequently, the usage of the new method will not be reported in the report file generated by RNASeqGUI. Future releases of RNASeqGUI will try to face this issue as well.

## 3. Conclusions

In this work, we have presented a novel version of RNASeqGUI that combines the flexibility of a graphical user interface with the tools available in Bioconductor for RR. The novel version significantly extends the original version with respect to several aspects [15, 17] (see Figure 1).

For each comprehensive analysis, not only does RNASeqGUI keep track of all actions executed by the user, but it also provides a set of cached objects saved in a database (by storing some intermediate results of the analysis) and in addition it generates a human readable report, which combines data, figures, and tables within the source code used to generate them. In this manner, the results (i.e., figures, tables, etc.) can be directly used in a publication, while the report can be viewed as a kind of supplementary information of a paper. Moreover, the database of cached objects can be shared via Internet allowing collaborators, reviewers, and readers to perform the same analysis and using the same data. Thanks to the report and thanks to the availability of cached objects database, not only does the user promote the transparency of his own work, but he also improves knowledge transfer and allows other readers to execute alternate analysis starting from intermediate results of the original analysis carried out.

Moreover, we extended RNASeqGUI in the number of interfaces and functionalities, also within each interface. We added the possibility of handling complex/multifactor designs by using several different DE methods and the possibility of conducting two different types of analyses for biological/technical replicates and also implemented the Pathway and Gene Ontology analysis. Therefore, the novel version constitutes a self-containing software able to support researchers in extracting biologically relevant information from the analysis of large datasets of RNA-Seq experiments. RNASeqGUI is a growing platform for the analysis of RNA-Seq data. Future releases will include other functionalities such as the possibility of identifying and estimating isoform abundances, in order to extend the range of supported features [17].

Finally, we aim that this work will constitute a proof of concept on how RR feature can be incorporated in GUIs in a useful and suitable way. Therefore, it will promote the development of novel computational software for the analysis of other NGS data (e.g., ChIP-Seq data, BS-Seq, etc.) in the spirit of RR.

## Supplementary Material

1) Supplementary Figure 1 shows how the report file is automatically written by RNASeqGUI in R-markdown format.
2) Supplementary Figure 2 describes how the report is showed via a web browser to the user after the clicking of the "Report" button in the main interface of RNASeqGUI.
3) Supplementary Figure 3 shows the code chunk that generates the Fold Change Plot depicted.
4) Supplementary Figure 4 shows the code chunk A that was executed during the generation of the boxplot and the code chunk B that was executed during the compilation of the report file from report.Rmd to report.html.

## Figures and Tables

**Table 1:** Time (in seconds) necessary to execute the counting procedure for RNA-Seq reads on two example datasets (*mouse* and *Drosophila* datasets). On the rows are represented two datasets used for the read counting step and the columns indicate if the parallel computing was used or not, on two different machines. The test was performed on a desktop personal computer with Intel I7-4790K@4.00 GHz and 24 GB of RAM, running Ubuntu 14.04 and on a Cluster node composed of 12 cores of Intel Xeon X5650@2.67 GHz, with 64 GB of RAM running CentOS release 6.5.

	Desktop		Cluster	
	Parallel (s)	Not parallel (s)	Parallel (s)	Not parallel (s)
*Mouse*	725	2027	2339	3409
*Drosophila*	442	559	416	969
