# Supplementary material for: Advancements in RNASeqGUI towards a Reproducible Analysis of RNA-Seq Experiments
Source: Biomed Res Int. 2016 Feb 10;2016:7972351. doi: 10.1155/2016/7972351 (PMC4764726; doi:10.1155/2016/7972351)

Supplementary data

Supplementary Material Figure 1

```
# The mm9 project report
### Project created the 2015-05-28 11:31:32

 * In the *NOISeq Interface*, you clicked the **Run NOISeq** button at `2015-05-28 11:36:02`
and the c_vs_dec.txt_prob=0.8_DE_genes_NOISeq.csv file has been saved in the `mm9\Results` folder.

You chose the following count file: `c_vs_dec.txt`, prob: `0.8`, Project: `mm9`,
Tissue= c('c','c','dec','dec'), TissueRun= c('S1','S5','S4','S8').

This R code has been run:

 ```{r}
require(NOISeq)
the.file2='c_vs_dec.txt'
noide2_db <- InitDb(db.name=paste(the.file2,'noide2_db',sep='_'), db.path='cache')
x <- LoadCachedObject(noide2_db, 'maindataframe_key')
the.file <- LoadCachedObject(noide2_db, 'the_file_key')
Project <- LoadCachedObject(noide2_db, 'project_key')
conditions <- LoadCachedObject(noide2_db, 'conditions_key')
TissueRuns <- LoadCachedObject(noide2_db, 'tissueruns_key')
p <- LoadCachedObject(noide2_db, 'p_key')
technical='TRUE'
print('You loaded this count file: ')
print(head(as.matrix(x)))
mynoiseq = NULL
if (technical == TRUE){ # technical replicate
      print('NOISeq has been started on TECHNICAL replicates')
     #myfactors = data.frame(Tissue = conditions, TissueRun = TissueRuns)
     #mydata <- NOISeq::readData(data=x, factors = myfactors)
     #mynoiseq = noiseq(mydata,k=0.5,norm='n',factor='Tissue',pnr = 0.2,nss = 5,
     #v = 0.02,lc = 0,replicates=technical)
      mynoiseq <- LoadCachedObject(noide2_db, 'mynoiseq_key')
  }else{ # biological replicate
     print('NOISeqBIO has been started on BIOLOGICAL replicates')
     #myfactors = data.frame(Tissue = conditions, TissueRun = TissueRuns)
     #mydata <- NOISeq::readData(data=x, factors=myfactors)
     #mynoiseq = noiseqbio(mydata, k = 0.5, norm = 'n', factor='Tissue', lc = 0, r = 20, adj = 1.5,
     #plot = FALSE, a0per = 0.9, random.seed = 12345, filter = 0)
      mynoiseq <- LoadCachedObject(noide2_db, 'mynoiseq_key')
  }
  print('First five lines of the results.')
  print( head(mynoiseq@results[[1]]) )
  #list_DE_NOISEQ = subset(mynoiseq@results[[1]], prob > p) # select significant genes
  list_DE_NOISEQ <- LoadCachedObject(noide2_db, 'listdenoiseq_key')
 ```
```

Supplementary Material Figure 2

RNASeqGUI_Projects/mm9/Logs/report.html

Search

The mm9 project report

Project created the 2015-05-28 11:31:32

- In the *NOISeq Interface*, you clicked the **Run NOISeq** button at `2015-05-28 11:36:02` and the c_vs_dec.txt_prob=0.8_DE_genes_NOISeq.csv file has been saved in the `mm9\Results` folder.

You chose the following count file: `c_vs_dec.txt`, prob: `0.8`, Project: `mm9`, Tissue= c('c', 'c', 'dec', 'dec'), TissueRun= c('S1', 'S5', 'S4', 'S8').

This R code has been run:

```
require(NOISeq)
the.file2='c_vs_dec.txt'
noide2_db <- InitDb(db.name=paste(the.file2,'noide2_db',sep='_'), db.path='cache')
x <- LoadCachedObject(noide2_db, 'maindataframe_key')
the.file <- LoadCachedObject(noide2_db, 'the_file_key')
Project <- LoadCachedObject(noide2_db, 'project_key')
conditions <- LoadCachedObject(noide2_db, 'conditions_key')
TissueRuns <- LoadCachedObject(noide2_db, 'tissueruns_key')
p <- LoadCachedObject(noide2_db, 'p_key')
technical='TRUE'
print('You loaded this count file: ')
```

```
## [1] "You loaded this count file: "
```

```
print(head(as.matrix(x)))
```

```
##                      S1   S5   S4   S8
## ENSMUSG00000063889 1935 1924 1404 1307
## ENSMUSG00000024231 1724 1639 1636 1674
## ENSMUSG00000024232  208  220  149  165
## ENSMUSG00000024235 1201 1241 1942 1843
## ENSMUSG00000024234  902  937  935  880
## ENSMUSG00000033960  613  683  615  618
```

```
mynoiseq = NULL
if (technical == TRUE){ # technical replicate
     print('NOISeq has been started on TECHNICAL replicates')
    #myfactors = data.frame(Tissue = conditions, TissueRun = TissueRuns)
    #mydata <- NOISeq::readData(data=x, factors = myfactors)
    #mynoiseq = noiseq(mydata,k=0.5,norm='n',factor='Tissue',pnr = 0.2,nss = 5,
    #v = 0.02,lc = 0,replicates=technical)
     mynoiseq <- LoadCachedObject(noide2_db, 'mynoiseq_key')
 }else{ # biological replicate
    print('NOISeqBIO has been started on BIOLOGICAL replicates')
    #myfactors = data.frame(Tissue = conditions, TissueRun = TissueRuns)
    #mydata <- NOISeq::readData(data=x, factors=myfactors)
    #mynoiseq = noiseqbio(mydata, k = 0.5, norm = 'n', factor='Tissue', lc = 0, r = 20, adj = 1.5,
    #plot = FALSE, a0per = 0.9, random.seed = 12345, filter = 0)
     mynoiseq <- LoadCachedObject(noide2_db, 'mynoiseq_key')
 }
```

```
## [1] "NOISeq has been started on TECHNICAL replicates"
```

Supplementary Material Figure 3

```
plot(log10(results_NoiSeq[,2] * results_NoiSeq[,1]),log10(results_NoiSeq[,2]/results_NoiSeq[,1]),col='bla
main=paste('PlotFC ',the.file2,sep=''), xlab=paste('log10( ', colnames(results_NoiSeq)[2],' * ',
colnames(results_NoiSeq)[1],')',sep=''),ylab=paste('log10( ',colnames(results_NoiSeq)[2],
' / ',colnames(results_NoiSeq)[1],' )',sep=''),pch=19,cex=0.3)
DE_genes_NoiSeq = subset(results_NoiSeq, prob>p)
points(log10(DE_genes_NoiSeq[,2] * DE_genes_NoiSeq[,1]), log10(DE_genes_NoiSeq[,2]/DE_genes_NoiSeq[,1]),
pch=19, col='red', cex=0.5)
```

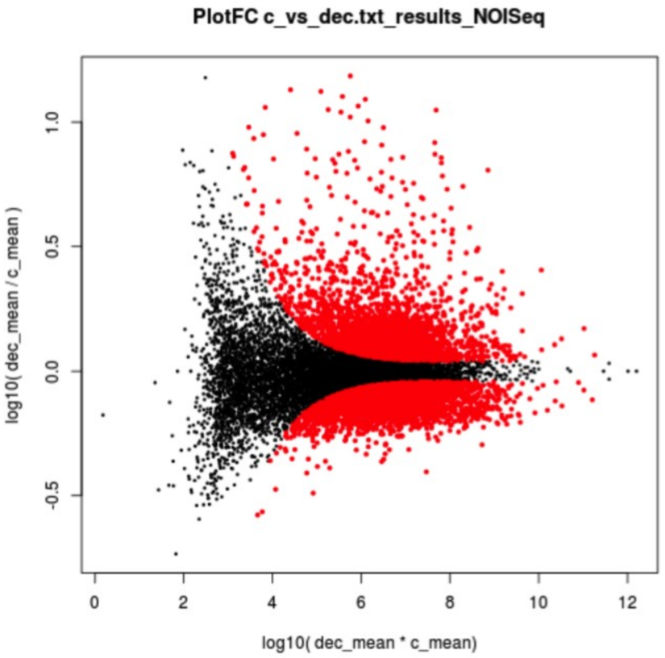

Supplementary Material Figure 4

Ⓐ
```
#x <- edgeR::DGEList(counts = x)
#x <- edgeR::calcNormFactors(x,method='upperquartile')
#x <- edgeR::estimateCommonDisp(x, verbose=FALSE)
#x <- edgeR::estimateTagwiseDisp(x)
#myuqua <- x$pseudo.counts
```
Ⓑ
```
myuqua <- LoadCachedObject(uqua.db, 'myuqua_key')
boxplot(log(myuqua+1),col=(c('red','blue','gold','darkgreen')), main='Upper Quartile BoxPlot',las=2)
```

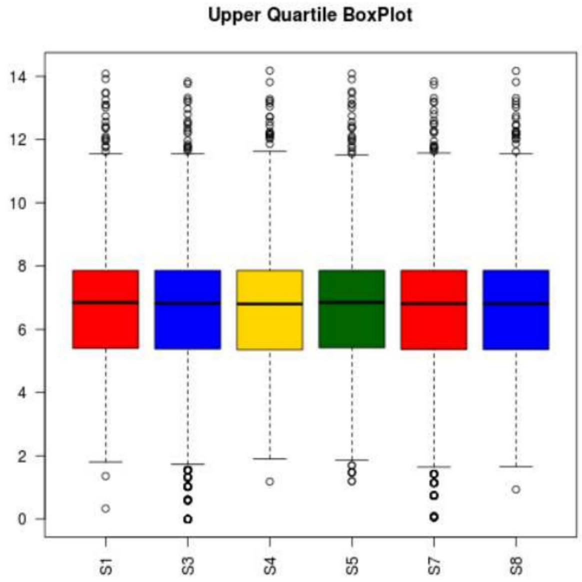

Supplement: Supplementary file 1 — 1) Supplementary Figure 1 shows how the report file is automatically written by RNASeqGUI in R-markdown format. 2) Supplementary Figure 2 describes how the report is showed via a web browser to the user after the clicking of the "Report" button in the main interface of RNASeqGUI. 3) Supplementary Figure 3 shows the code chunk that generates the Fold Change Plot depicted. 4) Supplementary Figure 4 shows the code chunk A that was executed during the generation of the boxplot and the code chunk B that was executed during the compilation of the report file from report.Rmd to report.html. [file 7972351.f1.pdf]
